# Category-Specific Semantic Impairment After Left Anterior Thalamic Infarction: A Case Report

**DOI:** 10.7759/cureus.102437

**Published:** 2026-01-27

**Authors:** Nobuhiro Takahashi, Mimpei Kawamura, Hiroki Tomita, Mamiko Sato, Yasutaka Kobayashi

**Affiliations:** 1 Department of Rehabilitation, Faculty of Health Science, Fukui Health Science University, Fukui, JPN; 2 Department of Medical Welfare, Faculty of Health Sciences, Kyoto Koka Women's University, Kyoto, JPN; 3 Department of Rehabilitation, Fukui General Clinic, Fukui, JPN

**Keywords:** anterior thalamic infarction, category-specific semantic impairment, ischemic stroke, nonverbal semantic judgment, odd-one-out task, semantic control, subcortical aphasia, thalamocortical semantic network

## Abstract

Thalamic lesions can result in a wide range of higher-order cognitive deficits; however, their association with category-specific semantic impairment has not been sufficiently examined. We report a 41-year-old man with a left anterior thalamic infarction who exhibited fluent speech with preserved repetition but showed asymmetric performance declines across semantic categories in both noun comprehension and naming, particularly under low-familiarity conditions. Detailed assessment revealed category-specific semantic impairment characterized by an interaction between semantic category and item familiarity, rather than a uniform semantic deficit. In addition, the patient demonstrated difficulty in a non-verbal semantic judgment task (Semantic Odd-One-Out Task), particularly when selecting among semantically close items, indicating impaired resolution of semantic competition. These findings suggest that the impairment was not limited to lexical retrieval processes but may extend to dysfunction in the regulatory (semantic control) mechanisms of the broader semantic processing network. This case provides clinically valuable evidence supporting the role of the thalamus as a key node in the control and integration of distributed semantic networks.

## Introduction

Thalamic lesions have long been recognized to cause a wide variety of higher-order cognitive impairments, including aphasia; however, their clinical manifestations often differ from those of cortical aphasia and are characterized by considerable heterogeneity [1,2]. Thalamic aphasia is frequently associated with relatively preserved speech fluency and repetition, alongside impaired word retrieval and instability in semantic processing, making it difficult to classify within a single aphasia subtype [1,2].

Recent studies have demonstrated that the thalamus functions not merely as a sensory relay nucleus but as a critical hub that modulates and integrates activity across distributed cortical networks, contributing to the regulatory aspects of language processing such as lexical retrieval, semantic selection, and conflict resolution [1,3-5]. From this perspective, language disturbances following thalamic lesions are increasingly understood not as the result of damage to specific semantic representations themselves, but rather as reflecting disrupted coordination within distributed cortical language networks. In simple terms, semantic memory depends on a distributed brain network that links word forms to conceptual knowledge, and successful language use requires not only storing meaning but also selecting the appropriate meaning in context. The thalamus is thought to support this process by coordinating communication between cortical regions, rather than by holding semantic representations itself.

In contrast, category-specific semantic impairment affecting word comprehension and lexical retrieval has been primarily investigated in association with cortical lesions since the seminal report of temporal lobe damage by Warrington and Shallice [6]. Category-specific semantic impairment refers to a selective difficulty in processing words from particular semantic categories (e.g., living things versus artifacts) that cannot be fully explained by general aphasia severity. Subsequent theoretical models have proposed that differences in the semantic features required for processing biological versus man-made categories-such as visual versus functional attributes-underlie category-specific effects [7-9]. These models largely assume that semantic representations are localized within cortical regions, whereas the contribution of subcortical structures to category-specific semantic processing has received comparatively little attention.

However, there have been very few reports examining whether similar category-specific semantic impairments can arise from thalamic lesions in the absence of overt cortical damage, and the underlying pathophysiological mechanisms remain poorly understood [2,10]. In particular, it remains unclear whether category-specific semantic impairment following thalamic lesions reflects disruption of semantic representations per se or impaired regulatory control over semantic processing.

The present case is characterized by a left anterior thalamic infarction in which asymmetric, category-specific impairments were observed across semantic categories, affecting both noun comprehension and naming, particularly under low-familiarity conditions, together with difficulty on a non-verbal semantic judgment task (Semantic Odd-One-Out Task). These findings suggest that the impairment extended beyond lexical retrieval processes and may reflect disruption of integrative and regulatory (semantic control) functions within the thalamocortical semantic network.

Although this case has previously been reported in a Japanese academic journal, the present report has been substantially revised and expanded for an international readership, incorporating additional clinical observations and theoretical considerations regarding semantic control and thalamocortical network function, and is presented here as an English-language case report.

## Case presentation

The patient was a 41-year-old right-handed man with no prior neurological history and independent activities of daily living before symptom onset. Seven days prior to presentation, he became unable to perform routine work procedures and experienced increasing difficulty understanding spoken language and written text. Because these symptoms persisted without improvement, he visited our hospital.

**Figure 1:**
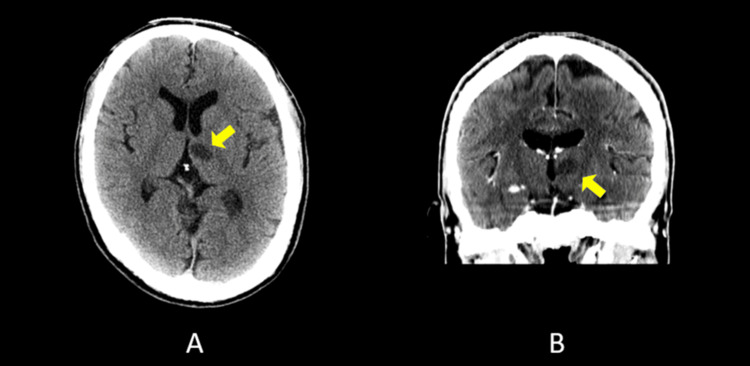
Non-contrast head CT obtained at admission showing a left anterior thalamic infarction Non-contrast head CT images obtained at admission demonstrate a focal low-density lesion in the left anterior thalamus (yellow arrows). Panel (A) shows an axial slice, and Panel (B) shows a coronal slice. No cortical lesions are observed. CT: computed tomography

Non-contrast head computed tomography revealed a focal low-density lesion in the left anterior thalamic region (Figure 1), and he was diagnosed with a left anterior thalamic infarction and admitted for treatment. No cortical lesions were identified. No abnormalities were observed on cranial nerve, motor, sensory, or cerebellar examinations. Two years earlier, the patient had undergone pacemaker implantation for complete atrioventricular block. At presentation, he was alert and fully conscious.

His speech was relatively fluent, with preserved sentence-level output and no articulatory distortion or phonemic paraphasia. However, his utterances contained few nouns and were characterized by frequent use of pronouns, interjections, and circumlocution, resulting in so-called “empty speech.” In conversational settings, overt comprehension deficits were not prominent. Anticoagulant therapy was initiated immediately after admission, and speech-language therapy began the following day.

Initial neuropsychological assessment was conducted between hospital days 2 and 22. On the Standard Language Test of Aphasia (SLTA) (Table 1) [11], single-word comprehension and repetition were well preserved. In contrast, the naming task revealed frequent semantic paraphasias, such as naming “deer” as “goat,” as well as circumlocutory responses. The patient frequently inserted interjections such as “uh” and expressed uncertainty with remarks such as “What do you call it?”, suggesting unstable lexical retrieval rather than articulatory or phonological impairment. Verbal fluency was markedly impaired, with only two words produced. During sentence comprehension tasks, he occasionally asked about word meanings within sentences, for example, “What does ‘iron bridge’ mean?”, indicating subtle instability in semantic access despite preserved overall comprehension.

**Table 1:** Results of the Standard Language Test of Aphasia (SLTA) Percentages indicate the proportion of correct responses for each subtest. Stage scores represent SLTA rating levels ranging from Stage 1 to Stage 6, with Stages 5 and 6 considered within the normal (correct) range. Word fluency indicates the number of animal names produced within the standard 60-second time limit of the SLTA.

Domain/subtest		Initial	Follow-up
I. Listening			
	Auditory word recognition	100%	100%
	Sentence comprehension	90%	100%
	Follow verbal commands	90%	90%
	Kana letter discrimination	100%	100%
II. Speaking			
	Object naming	70%	95%
	Word repetition	100%	100%
	Describe behaviors	90%	100%
	Picture story explanation	Stage 5	Stage 5
	Sentence repetition	100%	100%
	Word fluency	2 words	11 words
III. Reading			
	Kanji word–picture matching	100%	100%
	Kana word–picture matching	100%	100%
	Sentence–picture matching	80%	100%
	Follow written commands	90%	100%
IV. Writing			
	Write kanji words	100%	100%
	Write kana words	100%	100%
	Narrative writing	Stage 5	Stage 5
	Dictate kana letters	100%	100%
	Dictate kanji words	100%	100%
	Dictate kana words	100%	100%
	Dictate short sentences	60%	100%
V. Calculation			
	Calculation	80%	100%

To further evaluate lexical-semantic processing, the semantic category-based noun subtest of the Test of Lexical Processing in Aphasia (TLPA) [12] was administered. Asymmetric performance differences across semantic categories were observed in both auditory comprehension and naming, particularly under low-familiarity conditions (Table 2). Notably, performance in the plant category was markedly impaired. Analysis of naming errors revealed that semantic paraphasias were the most frequent error type, accounting for more than half of all errors, whereas phonological errors were rare (Table 3).

**Table 2:** Results of the semantic category-specific noun test of the Test of Lexical Processing in Aphasia (TLPA): accuracy and z-scores by semantic category Percentages indicate the proportion of correct responses. z-scores were calculated based on reference data provided in the TLPA manual (aphasic population), reflecting relative performance within an aphasia cohort rather than deviation from healthy controls.

Semantic category	High-familiarity words				Low-familiarity words			
	Initial (%)	z-score	Follow-up (%)	z-score	Initial (%)	z-score	Follow-up (%)	z-score
Indoor locations	80	0.04	80	0.04	50	−0.64	70	0.13
Buildings	80	−0.05	100	0.88	60	0.06	70	0.43
Vehicles	90	0.22	100	0.76	60	−0.05	60	−0.05
Tools	100	0.7	100	0.7	60	0.05	70	0.41
Processed foods	80	0.09	90	0.58	40	−0.60	80	0.96
Fruits and vegetables	70	−1.08	90	0.13	40	−1.15	60	−0.30
Plants	70	−0.29	90	0.66	0	−2.04	10	−1.67
Animals	90	0.11	90	0.11	60	−0.24	70	0.15
Body parts	100	0.7	100	0.7	60	0.06	50	−0.37
Colors	90	0.55	80	0.03	70	0.62	80	1.02
Mean	85	0.12	92	0.59	50	−0.48	62	0.09

**Table 3:** Error types in the TLPA naming task Percentages indicate the proportion of each error type relative to the total number of naming errors. TLPA: Test of Lexical Processing in Aphasia

Error type	Initial (No. of errors)	Initial (%)	Follow-up (No. of errors)	Follow-up (%)
Semantic paraphasia	40	61.54	33	71.74
Circumlocution	9	13.85	3	6.52
No response	8	12.31	8	17.39
Unrelated error	4	6.15	0	0
Other	4	6.15	2	4.35
Total errors	65	100	46	100

**Figure 2:**
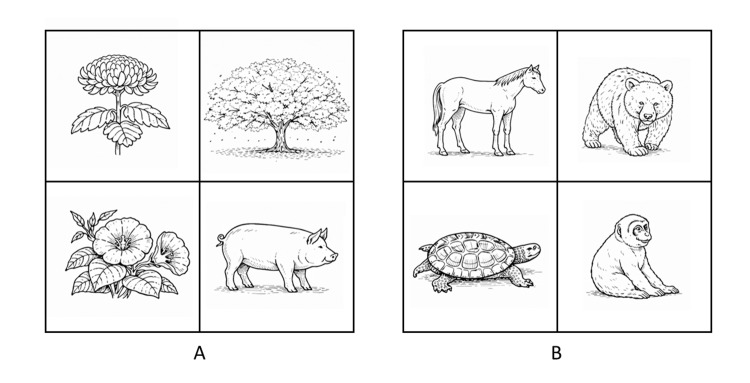
Examples of the Semantic Odd-One-Out Task Examples of the Semantic Odd-One-Out Task used to assess non-verbal semantic judgment. The task required the selection of one item that was semantically different from the others among simultaneously presented line-drawing stimuli. Panel (A) illustrates the distant category condition, in which the semantic categories of the stimuli were clearly distinct, whereas Panel (B) illustrates the close category condition, in which the semantic similarity among the stimuli was high. Detailed performance results are shown in Table 4. Image credits: the authors

To assess non-verbal semantic judgment, a Semantic Odd-One-Out Task developed by the authors was administered (Figure 2, Table 4). While performance was high in the distant-category condition, accuracy declined in the close-category condition, indicating difficulty selecting among semantically similar alternatives.

**Table 4:** Results of the Semantic Odd-One-Out Task The Semantic Odd-One-Out Task assesses non-verbal semantic judgment by requiring the selection of the item that is semantically different from the others among simultaneously presented stimuli. The distant category condition involves stimuli with clearly distinct semantic categories, whereas the close category condition involves stimuli with high semantic similarity, requiring resolution of semantic competition.

Condition	Initial (correct/total)	Accuracy (%)	Follow-up (correct/total)	Accuracy (%)
Distant category	38/40	95	40/40	100
Close category	26/40	65	34/40	85

Visual and performance-based abilities were further evaluated using the Wechsler Adult Intelligence Scale-Third Edition (WAIS-III) (Table 5) [13]. Although the Perceptual Organization Index was relatively preserved, variability across subtests was observed, with lower scores particularly on tasks involving meaningful visual stimuli, such as Picture Arrangement and Picture Completion.

**Table 5:** Results of the Wechsler Adult Intelligence Scale-Third Edition (WAIS-III) Index scores are age-adjusted standard scores (mean = 100, SD = 15). Subtest scores are age-adjusted scaled scores (mean = 10, SD = 3).

Domain	Measure	Initial	Follow-up
Index scores	Performance IQ (PIQ)	72	83
	Perceptual Organization Index (PO)	87	97
	Processing Speed Index (PS)	66	69
Subtests	Picture Arrangement	3	5
	Picture Completion	3	6
	Object Assembly	6	7
	Block Design	11	12
	Matrix Reasoning	10	11
	Digit Symbol-Coding	3	4
	Symbol Search	5	5

Re-evaluation was conducted between hospital days 39 and 40. On the SLTA, improvement was observed across multiple subtests, and naming performance showed substantial recovery (Table 1). However, category-specific performance differences on the TLPA semantic category-based noun test persisted, especially in the low-familiarity plant category (Table 2). Error analysis of naming responses continued to show semantic paraphasia as the predominant error type (Table 3). On the Semantic Odd-One-Out Task, performance in the close-category condition improved, although residual errors were observed, whereas performance in the distant-category condition remained high (Table 4). On the WAIS-III, the Perceptual Organization Index improved to within the normal range, while inter-subtest variability persisted, suggesting selective vulnerability of tasks requiring semantic processing (Table 5).

## Discussion

This case is characterized by the presence of category-specific semantic impairment affecting both noun comprehension and naming following a left anterior thalamic lesion. Since the seminal report of temporal lobe damage by Warrington and Shallice [6], category-specific semantic impairment has been primarily understood as a phenomenon associated with cortical lesions. Subsequent theoretical models have proposed that differences in the semantic features required for processing biological versus man-made categories-such as visual versus functional attributes-underlie category-specific effects [7-9]. These models generally assume that semantic representations are distributed within cortical regions, while the contribution of subcortical structures, particularly the thalamus, has been largely underemphasized. Therefore, the observation of comparable category-specific semantic impairment in a thalamic lesion without evident cortical involvement suggests that category specificity may not be fully explained solely by cortical localization of semantic representations.

Recent neuroscientific research has increasingly emphasized the role of the thalamus not merely as a sensory relay nucleus but as a critical hub that modulates and integrates activity across distributed cortical networks [3,4]. In particular, the anterior thalamic nuclei are extensively connected with frontal and temporal cortical regions and are thought to contribute to regulatory aspects of semantic processing, including semantic retrieval, selection, and conflict resolution [4,5]. From this perspective, language impairment following thalamic lesions may be better understood not as damage to semantic representations per se, but rather as potentially reflecting disrupted coordination and control within distributed cortical semantic networks.

In the present case, speech was fluent, phonemic paraphasia was absent, and repetition was well preserved. In addition, spontaneous inquiries about word meanings, such as “What does ‘iron bridge’ mean?”, resemble features observed in transcortical sensory aphasia. However, word comprehension deficits were subtle and not detected by the SLTA, nor did they prominently affect everyday conversation, making it difficult to classify this case as typical transcortical sensory aphasia. Instead, detailed assessment using the TLPA and spontaneous questioning about word meanings suggested unstable access to semantic representations rather than a loss of semantic knowledge itself. Thus, in addition to impaired access from semantic representations to lexical forms (anomia), the findings may indicate that partial impairment of access from lexical input to semantic representations was also present, resulting in an imbalance affecting both comprehension and production. This pattern is consistent with the concept of bidirectional anomia, in which both input and output pathways to semantic representations are disrupted [14,15].

This interpretation is further supported by the test profile. While the SLTA comprehension tasks involving a small number of mixed-category choices failed to detect impairment, the TLPA semantic category-based noun test-requiring identification of a target word from multiple items within the same category-revealed clear comprehension deficits. This suggests that although coarse, category-level semantic access was relatively preserved, lexical retrieval and semantic matching within categories, particularly when resolving competition among semantically similar items, were impaired. Consistently, reduced performance in the close-category condition of the non-verbal Semantic Odd-One-Out Task indicates difficulty in selecting and inhibiting closely related semantic alternatives. Such impairments in resolving semantic competition are considered characteristic of dysfunction within the semantic control network [16,17], which is consistent with the interpretation that the anterior thalamic lesion in this case may have disrupted regulatory mechanisms governing semantic selection and competition.

Furthermore, performance on the WAIS-III showed relatively preserved scores on tasks using meaningless stimuli, whereas tasks involving meaningful visual stimuli were impaired. This dissociation suggests that vulnerability of a non-verbal semantic system could have contributed, at least in part, to the observed language deficits. Such findings are consistent with the view that semantic processing relies on distributed networks spanning both verbal and non-verbal domains, and that disruption of network-level regulation can manifest across modalities [4,16].

Compared with hemorrhagic lesions, thalamic infarctions are generally considered to be more focal and less likely to exert secondary effects on surrounding structures due to mass effect or hematoma-related compression. In contrast, thalamic hemorrhages often involve multiple thalamic nuclei and adjacent structures and are frequently associated with mass effect [18]. Because the present case involved an ischemic anterior thalamic infarction, it provided relatively favorable conditions for examining the relationship between thalamic function and clinical symptoms without substantial confounding effects from widespread structural compression.

This report has several limitations. First, as a single-case study, caution is required when generalizing the findings. Second, because the patient had an implanted pacemaker, magnetic resonance imaging could not be performed, precluding functional or detailed structural network analyses. Third, the Semantic Odd-One-Out Task used to assess non-verbal semantic judgment did not strictly control semantic categories across stimuli. A task design with stricter category control might have allowed more detailed examination of category-specific difficulties within the semantic system. Nevertheless, a major strength of this report lies in its comprehensive evaluation of both verbal and non-verbal semantic processing, enabling a multifaceted characterization of category-specific semantic impairment associated with thalamic lesions.

Overall, this case provides clinical evidence that anterior thalamic lesions can be associated with disruption of the regulatory (semantic control) functions of semantic processing networks, leading to category-specific semantic impairment in the absence of overt cortical damage.

## Conclusions

This case suggests that a left anterior thalamic lesion may be associated with category-specific semantic impairment affecting both noun comprehension and naming, even in the absence of overt cortical damage. Furthermore, the presence of difficulties in non-verbal semantic judgment, particularly when resolving competition among semantically similar alternatives, supports the interpretation that the observed deficit was not limited to lexical retrieval processes. Rather, these findings are consistent with disruption of the regulatory (semantic control) functions within a broader thalamocortical semantic network. Taken together, this case provides clinically meaningful evidence supporting the view that the thalamus may contribute to the control and integration of distributed semantic processing networks, with potential relevance to category-specific semantic performance across both verbal and non-verbal domains.
